# Transtheoretical-Based Model of Intervention for Diabetes and Prediabetes: A Scoping Review

**DOI:** 10.1155/2024/2935795

**Published:** 2024-04-29

**Authors:** Daniel Miezah, Mustapha Amoadu, Priscilla Nyamekye Opoku, Enoch Mensah Junior, Jethro Zutah, Paul Obeng, Jacob Owusu Sarfo

**Affiliations:** ^1^Department of Education and Psychology, University of Cape Coast, Cape Coast, Ghana; ^2^Department of Health, Physical Education and Recreation, University of Cape Coast, Cape Coast, Ghana

## Abstract

Diabetes is considered a public health problem worldwide, fostered by population growth, an increase in the overall quality of life, changes in dietary patterns, modifications in lifestyle habits, and the natural process of getting older. To properly control diabetes, the transtheoretical model (TTM) may be useful. This scoping review is aimed at identifying TTM interventions for diabetes. The study followed Arksey and O'Malley's six steps in conducting the scoping review. Four main databases (PubMed, Central, JSTOR, and ScienceDirect), Google Scholar, Google, and a reference list of identified articles were searched for literature. The study included peer-reviewed articles published online from 2000 to 2023 and published in the English language. At the end of the search, 3,514 entries were found in the four main databases, and 23 records were identified through Google, Google Scholar searches, and reference lists. After a thorough screening, 22 records were used for this review. The study found that the primary interventions based on the TTM for managing diabetes and prediabetes were educational materials to promote physical activity among diabetes and prediabetes individuals, health education, exercise, motivational interviews, self-tracking, and dietary changes. Further interventions on diabetes and prediabetes management could adopt the identified transtheoretical interventions to improve the health of their patients.

## 1. Introduction

Diabetes is considered a public health problem worldwide, fostered by population increase, an elevated standard of living, dietary shifts, lifestyle alterations, and ageing [1]. The continuous rise in the incidence of type 2 diabetes mellitus (DM) has contributed significantly to the substantial increase in the prevalence of DM. During the past decade, over 537 million adults (20–79 years old) are estimated to have diabetes worldwide [2]. According to the International Diabetes Federation [2], this number could rise to 643 million by 2030 and 783 million by 2045. Globally, over 6.7 million people died from diabetes in 2021 [3, 4]. Nonetheless, blood glucose levels can be controlled to greatly slow the onset of diabetes and its consequences through appropriate self-management practices [2].

Self-management of chronic diseases lowers the toll that diseases take on patients and enhances the effectiveness of treatment [5]. Self-management education for diabetic patients improves treatment outcomes and delays chronic consequences [6]. The conventional management of diabetes comprises regular visits by a qualified healthcare clinician, medication compliance, and lifestyle modification [7]. Given that the burden of the disease is on the increase and consequently affecting the younger population, the need to curb the situation cannot be overemphasized. The transtheoretical model (TTM) may be useful for properly controlling diabetes. Healthcare workers may be better prepared to assist positive and long-lasting behavioral changes if they are knowledgeable about the stages of behavior change and stage-specific interventions [6]. Hence, this scoping review mapped evidence on TTM intervention for diabetes management.

## 2. Methods

This review followed the recommended guidelines of Arksey and O'Malley [8] to conduct a scoping review. These guidelines involve defining the research questions, identifying relevant studies, selecting studies, collecting data, summarizing and synthesizing results, and embarking on consultation [8]. The research question that guided this study was the following: what are the available TTM interventions for DM and prediabetes management?

### 2.1. Identifying Relevant Studies

A literature search was conducted in four databases, including PubMed, Central, JSTOR, and ScienceDirect. The researchers focused on the four databases because they were readily accessible. Also, the authors conducted further searches in Google, Google Scholar, and Dimensions AI to supplement the four databases search. Using Medical Subject Headings (MeSH), searches were conducted in PubMed and later modified to search through Central, JSTOR, and ScienceDirect. Key terms and Boolean operators were also used to search through the four databases for relevant articles (see Table 1 for key terms and search strategy). Additional records were found by checking the reference lists of eligible papers. The latest search was done on March 16, 2023. Three authors (P.N.O., E.M.J., and J.Z.) collaborated with a chartered librarian at the Sam Jonah Library, University of Cape Coast, to conduct a thorough literature search, ensuring the identification of ample relevant literature.

### 2.2. Selection of Studies

**Figure 1:**
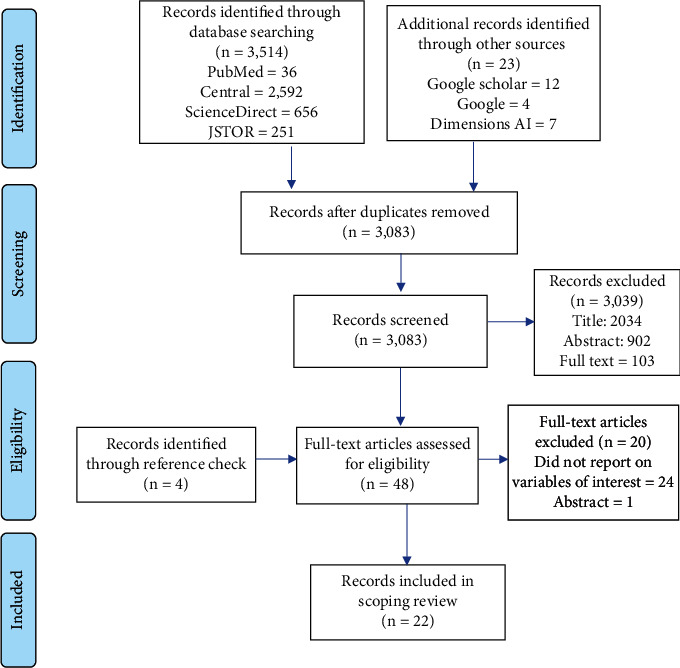
PRISMA flow chart of the records and screening process.

The selection of studies involved a thorough screening process against the current study's eligibility criteria, conducted independently by three authors (E.M.J., P.O., and J.Z.). This screening comprised three main steps. First, the titles and abstracts of the identified records were assessed for relevance. Second, relevant studies underwent a full-text review. Lastly, the full-text records were screened against the eligibility criteria, with studies meeting the inclusion criteria selected for data extraction. The authors convened to compare their screening outcomes and resolve any discrepancies. In cases of conflict, a fourth author (P.O.) was consulted to facilitate resolution.  Figure 1 illustrates the screening process for the identified studies.

### 2.3. Data Extraction and Charting

Data were extracted individually by E.M.J., P.O., and J.Z. and analyzed by M.A. The data collected included the authors, year, country, study design, population, sample size, facilitators, barriers, interventions, and outcomes. All the authors resolved disagreements during data collection at meetings. To guarantee the validity and completeness of the data for this scoping review, an expert in the subject area was consulted. Finally, all authors reviewed and approved the extracted data before conducting data synthesis and analysis.

### 2.4. Data Synthesis and Analysis

The authors conducted a qualitative synthesis of the extracted data. In conducting the synthesis, three authors (E.M.J., P.O., and J.Z.) read through the extracted data three times individually to familiarize themselves with the data and also to understand the extracted data while taking note of recurrent terms, themes, and subthemes. The three authors came together to read through the extracted data two times and identified recurrent terms, themes, and subthemes. The identified themes and subthemes were described to form the findings of the study. Also, the characteristics of the studies, such as the study designs and the type of study population, were presented using a bar graph. Again, the countries where the extracted studies were conducted were presented on a map. An independent researcher (M.A.) reviewed the results and approved them before discussing them.

### 2.5. Consultations

The authors embarked on consultation at every stage of the work. For instance, an expert in diabetes research (a senior lecturer at the Department of Health, Physical Education and Recreation, University of Cape Coast) reviewed the introduction of the protocol and approved it before proceeding with the method section of the protocol. He also reviewed and approved the method and the entire work before submission to the journal. Again, a chartered librarian at the Sam Jonah Library was consulted to retrieve additional records for the study, and the search strategy used in the current review was approved.

## 3. Results

At the end of the search, 3,514 entries were found in the four main databases (PubMed, Central, JSTOR, and ScienceDirect). Twenty-three records were also found through Google and Google Scholar searches. Three thousand eighty-three records underwent additional screening after a total of 454 duplicate records were eliminated. Forty-eight full-text records were carefully screened, and their eligibility was evaluated following the standards shown in Table 1. Finally, this scoping review covered 22 records.  Figure 1 displays specifics about the screening procedure and search results.

### 3.1. Characteristics of the Included Studies

**Figure 2:**
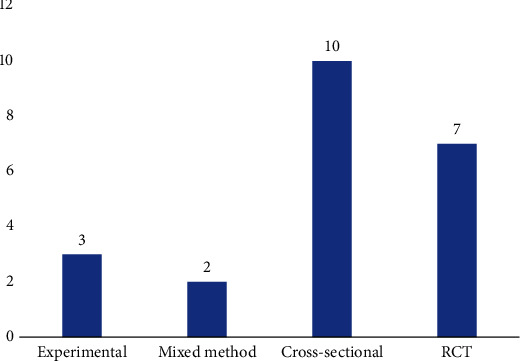
Research design explored by the included studies.

**Figure 3:**
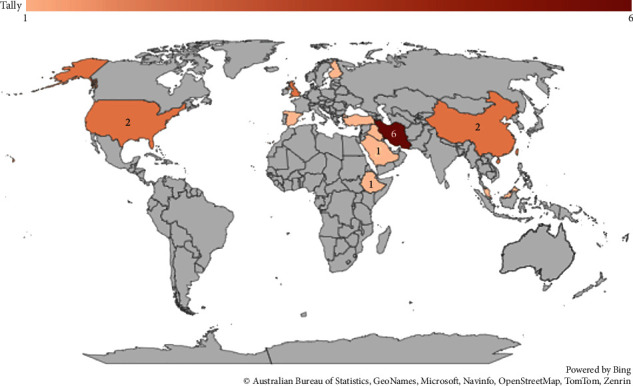
Map displaying countries where the research was done.

The majority of the included studies were cross-sectional surveys (10), followed by randomized controlled trials (RCT) (7) and experimental research designs (3) (see Figure 2). Also, the majority of the studies were conducted in Iran (6), followed by the United Kingdom (2), China (2), the United States (2), and Taiwan (2) (see Figure 3). The details of the extracted data are presented in Table S1.

### 3.2. Main Findings

Reviewed studies found that educational content, health education, exercise, motivational interviews, self-monitoring, and dieting are the main TTM interventions used in diabetes management.

#### 3.2.1. Educational Contents

One effective way to increase the level of physical activity among diabetic patients is to use educational content [9]. Also, educational content based on the TTM is useful for increasing the self-efficacy of patients to engage in physical activities [10]. Moreover, educational content based on the TTM caused progress and promotion in the physical activity of patients' children [11]. Using educational content to promote behavior modification in prediabetics is expected to delay or prevent the development of type 2 diabetes in Saudi Arabia [12]. Specifically, health education combined with the TTM of behavior change helped to alleviate the negative psychological state of patients, enhance adherence to fluid intake, and efficiently decrease the occurrence of acute complications related to dialysis [13]. A study [14] showed that dietary knowledge significantly motivated participants to progress to the later stages of behavior change, which improved the outcome of glycemic control. However, low health literacy was significantly associated with worse glycemic control.

#### 3.2.2. Physical Activity

Pros and cons of exercise, self-efficacy, counterconditioning, and self-liberation were predictors of physical activity behavior [15]. The study also revealed that the best predictors of physical activity among diabetic patients were self-efficacy and the change process (consciousness raising, dramatic relief, environmental re-evaluation, self-re-evaluation, social liberation, counterconditioning, helping relationships reinforcement management, self-liberation, and stimulus control). There was a positive correlation between high levels of education and increasing physical activity levels; that is, the level of education can serve as both a facilitator and a barrier to physical activity. The study showed reduced physical activity with increasing age. That is, there is a negative correlation between physical activity and age [15]. Moreover, a significant correlation was found between the mean daily exercise and self-efficacy, and TTM-based interventions were effective in promoting and maintaining exercise behavior [16].

#### 3.2.3. Motivational Interview

A study by [17] identified four major themes regarding adherence to lifestyle change (stumbling block, self-care belief, knowledge implementation, and self-empowerment) and advised healthcare providers to identify patients' stages of change before implementing lifestyle change. Also, Selçuk-Tosun and Zincir [18] revealed that the TTM-based motivational interview method increased the self-efficacy level of participants with type 2 DM, which helped them improve their metabolic control and health behavior stages over 6 months. Furthermore, a specialist dietitian with training in motivational interview and behavioral change can successfully provide a TTM intervention to people with diabetes, resulting in increased physical activity and stage of change revealed by [19].

#### 3.2.4. Self-Monitoring

With regular exercise and diet management, social support scores were significantly higher in the later stages of behavior change than in the earlier stages. Similarly, the results for self-efficacy and compliance in blood glucose monitoring, regular exercise, and diet control were significantly higher in the later stages of behavior change compared to the earlier stages [20]. A study by [21] displayed that the TTM enhances resources, supports self-management, and helps people develop effective diabetic self-management techniques. According to [22], research, planning, belief in diabetes, support from the treatment team, understanding of nutrition, and religious views help self-management in the preaction and action stages. The barriers to self-management in the action and preaction stages were lower socioeconomic status, poor performance of the treatment team, physical–intellectual factors, and lack of planning to change. Moreover, according to [23], managing diabetes requires a motivated client and skilled healthcare provider. Healthcare professionals who possess knowledge about the behavior change process and stage-specific interventions may be well equipped to facilitate positive and lasting behavior changes.

#### 3.2.5. Dieting

A study conducted by [20] revealed a statistically significant difference between the experimental and control group scores on knowledge of diabetes diet, behavior of diabetes diet, and the quality of life in prediabetes patients. The study also confirmed that dietary intervention based on TTM can effectively improve knowledge of diabetes diet, behavior of diabetes diet, and the quality of life in patients with prediabetes.

## 4. Discussion

This scoping review is aimed at mapping the evidence of TTM intervention for diabetes management in terms of target behaviors, its facilitators, and barriers. We found that educational content, health education, exercise, dieting, self-monitoring, and motivational interviews are the main interventions for diabetes management based on the TTM of behavior modification.

### 4.1. Exercise

We observed that regular exercise is strongly linked to an increase in self-confidence [15, 16]. Various factors within the process of change help to facilitate an increase in confidence, such as becoming more aware of one's habits (consciousness raising), thinking about the impact of the environment on behavior (environmental re-evaluation), and taking time to self-reflect (self-re-evaluation) [16]. Additionally, external factors such as societal attitudes towards physical activity, the creation of new habits (counterconditioning), positive reinforcements, and being mindful of triggers (stimulus control) all contribute to an individual's level of self-efficacy [15, 16].

In regard to diabetic patients specifically, self-efficacy and the process of change were the strongest predictors of whether or not these individuals engage in physical activity [16]. Those who engaged in regular exercise routines were found to have higher levels of self-efficacy and a stronger belief in the positive impact exercising had on their health [15, 16, 18]. Conversely, individuals with higher levels of self-efficacy had more control over their choices and the motivation to engage in physical activity [18]. Diabetic patients who initially struggled with exercising regularly may be due to a lack of confidence in their ability. Through exercising consistently and implementing strategies to maintain motivation, like finding an exercise buddy, and adjusting their environment to make exercise more accessible, they may begin to experience increased levels of self-efficacy. This newfound confidence may have led to a greater sense of control over their health choices and a continued commitment to regular physical activity [16].

### 4.2. Motivational Interview

The motivational interview method that was based on the TTM helped increase the level of self-confidence among individuals with type 2 diabetes. This, in turn, led to improvements in both their metabolic control and overall health behaviors. Through these interviews, patients were able to identify which stage of change they were in and received personalized recommendations for lifestyle modifications [17].

This review revealed four key important themes for ensuring adherence to lifestyle changes, particularly in the preaction and action stages. These themes included recognizing potential stumbling blocks, developing a strong belief in one's ability to care for oneself, implementing the knowledge gained from healthcare professionals, and empowering oneself to take control of a person's health [18]. Individuals with type 2 diabetes who were in the preaction stage struggled with making lifestyle changes. However, through the motivational interview based on the TTM, they could identify what was holding them back and understand why the recommended lifestyle changes were important [19]. Armed with this knowledge, they began implementing small changes in their daily routine, such as taking the stairs instead of the elevator or walking for 10 min after dinner. Gradually, as they built up their self-efficacy and saw positive results, they felt more empowered to take on bigger changes, such as incorporating regular exercise into their routine or making dietary changes.

### 4.3. Self-Monitoring

Self-monitoring as an intervention helped individuals with diabetes to adopt healthier self-management behaviors. This included regular exercise, controlling one's diet, ensuring compliance with blood glucose monitoring, and building self-efficacy [22]. However, some barriers to self-monitoring were realized in both the preaction and action stages. Barriers include lower socioeconomic status, physical and intellectual limitations, and a lack of planning for change [22, 23]. These barriers had a negative impact on self-efficacy levels and compliance. Diabetic patients who struggled to manage their blood glucose levels were encouraged to start using self-monitoring as a way to track their progress [23]. They began by checking their blood glucose levels at specified times throughout the day and logging the results.

### 4.4. Dieting

The patient's understanding of diabetes diets, dietary behaviors, and overall quality of life is a critical component of successfully managing diabetes. By using dietary interventions based on the TTM, patients with prediabetes improved their knowledge and behavior around diabetes diets, which led to an improved quality of life and overall well-being [20]. Patients who had been diagnosed with prediabetes struggled with making dietary changes and understanding what foods are best for their health. Through an intervention based on the TTM, the patients could learn about their current dietary behaviors and identify their stages of change [20]. With this knowledge, patients worked with healthcare professionals to create an individualized plan for dietary change that considers their preferences and goals. This included replacing high-carbohydrate snacks with more nutritious options or increasing vegetable intake.

### 4.5. Educational Content

Educational content is a major intervention in diabetes management using the transtheoretical behavior modification model. Educational materials effectively changed patients' belief systems, increased self-efficacy, educated patients on the importance of physical activity to overall health, and improved patients' compliance levels [9], [11–13]. Additionally, educational content served as motivation for patients to make lifestyle changes that enhanced their overall well-being. Patients who had been diagnosed with diabetes were motivated to learn more about the disease and its management [12]. They were provided with educational content that explained the importance of blood sugar monitoring, diet control, regular exercise, and other treatment options [11, 13]. As they learned more about diabetes, they felt more empowered to make the necessary changes to manage the disease effectively.

Educational content also provided the patients with the knowledge and tools they needed to maintain a healthy lifestyle [9]. Contents were in various forms, such as videos, pamphlets, or lectures. Patients who understood how their actions impacted their health were more likely to make sustainable changes. For instance, patients who struggled to control their blood sugar levels were admonished to learn about the importance of regular exercise in lowering blood sugar levels. Armed with this knowledge, they were motivated to start a daily exercise routine to improve their health [9]. Additionally, implementing educational content as part of a comprehensive diabetes management plan can help patients build knowledge, increase their self-efficacy, and facilitate lasting lifestyle changes that lead to better outcomes.

### 4.6. Recommendations for Practice

Healthcare professionals should employ tailored educational interventions rooted in the TTM to address the diverse needs and readiness levels of individuals with diabetes. These interventions should focus on enhancing knowledge about diabetes management, fostering self-efficacy, and promoting healthy behaviors like regular physical activity and dietary modifications. Incorporating motivational interviewing techniques into diabetes care can further boost patients' self-confidence and motivation to adopt lifestyle changes. Supporting individuals with diabetes in regular self-monitoring of blood glucose levels, physical activity, and dietary intake is essential, along with providing tools and resources for tracking progress. Structured exercise programs tailored to individual preferences and readiness levels should be developed to underscore the benefits of physical activity for glycemic control and overall well-being. Additionally, implementing dietary interventions grounded in the TTM can improve patients' understanding of diabetes diets and empower them to make healthier food choices. A multidisciplinary approach involving collaboration among healthcare professionals is crucial to ensure comprehensive support for patients across all stages of behavior change and address the multifaceted aspects of diabetes management. By integrating these recommendations into clinical practice, healthcare providers can effectively enhance diabetes management interventions and support individuals with diabetes in achieving optimal health outcomes.

### 4.7. Gaps in Literature and Recommendations for Future Research

Future research on the utilization of the TTM for diabetes management should focus on several key areas. First, there is a need to investigate how the model can be effectively utilized for vulnerable populations with diabetes, such as individuals who are overweight or those with other chronic diseases. Second, studies should examine how patient and healthcare provider characteristics, such as age, gender, education, health literacy, and comorbidities, impact diabetes management using the TTM intervention.

Additionally, future studies should focus on the target behaviors the intervention is aimed at improving, including diet, exercise, medication adherence, and self-monitoring of blood glucose levels. Furthermore, studies should employ reliable measures and adjust for confounding variables, particularly through RCT, to determine the specific behaviors that contribute to type 2 diabetes and the TTM-based interventions that are effective.

Finally, there is a need for longitudinal investigation into the long-term outcomes of the intervention. While some studies have reported positive short-term results, there is limited evidence on whether these effects are sustained over time. Addressing these areas through further research could help to optimize the TTM intervention for diabetes management.

### 4.8. Limitation of This Review

This review is subject to several limitations. Notably, there was a lack of consideration for confounding variables in most of the studies analyzed. Consequently, the findings may not fully account for all potential factors influencing the outcomes. The predominance of cross-sectional survey designs among the reviewed studies poses a significant limitation. Such designs are susceptible to response bias and may lack the ability to establish causality, limiting the generalizability of the findings. Another limitation stems from the inclusion criterion of English language publications only. This may have resulted in the exclusion of relevant literature published in other languages, potentially biasing the findings and conclusions.

Additionally, the review only included full-text articles, excluding studies behind subscription paywalls. This selection criterion may have led to the omission of pertinent literature, thereby limiting the comprehensiveness of the review. Also, studies behind paywalls were excluded, potentially excluding relevant research that could have contributed to a more comprehensive understanding of the topic. Despite these limitations, the review utilized a rigorous search strategy to identify relevant evidence, aiming to inform practice, policy, and future research endeavors.

## 5. Conclusion

Based on the findings of this scoping review, it is evident that interventions based on the TTM offer promising strategies for managing diabetes and prediabetes. The identified interventions, including educational materials, health education, exercise, motivational interviews, self-tracking, and dietary changes, align with the principles of the TTM, which emphasizes stages of change and processes of change. These interventions have the potential to address the complex behavioral and lifestyle factors associated with diabetes and prediabetes, promoting positive health outcomes and enhancing self-management practices among affected individuals.

However, while this review provides valuable insights into existing interventions, there remains a need for further systematic review and evaluation of the effectiveness of transtheoretical-based interventions in diabetes management. Future research should focus on synthesizing and analyzing empirical evidence from RCT, cohort studies, and intervention studies to ascertain the impact of transtheoretical interventions on glycemic control, adherence to treatment regimens, and overall health outcomes among individuals with diabetes and prediabetes.

Additionally, exploring the contextual factors influencing the implementation and uptake of transtheoretical-based interventions in diverse populations and healthcare settings is essential. Understanding the facilitators and barriers to intervention implementation can inform the development of tailored and culturally appropriate strategies to enhance the effectiveness and scalability of diabetes management programs.

## Figures and Tables

**Table 1:** Search strategy for papers on transtheoretical model intervention on diabetes management, target behaviors, facilitators, and barriers.

**Item**	**Search strategy**
Databases	PubMed, Central, ScienceDirect, JSTOR

Language	English

Time filter	2000–2023

Keywords	1. “Trans-theoretical Model” OR “Behaviour Change Model” OR “Behaviour Modification Model” OR “Trans-theoretical Model of Intervention”. 2. “Intervention” OR “Treatment” OR “Management” 3. “Diabetes Mellitus” OR “Type 2 Diabetes” OR “Diabetes”

Search strategy	#1 AND #2 AND #3 NOT animal

Inclusion criteria	The paper should be as follows: 1. Peer-reviewed literature 2. Published online from January 2000 to March 2023 3. Published in the English language 4. On transtheoretical model intervention in diabetes management, the target behaviors, facilitators, and barriers

Exclusion criteria	The paper should be as follows: Reviews, abstracts, commentaries, letters to editors, literature reviews

## Data Availability

All data generated or analyzed during this study are included in this published article (and its supporting information files).
