# Supplementary material for: Transtheoretical-Based Model of Intervention for Diabetes and Prediabetes: A Scoping Review
Source: J Diabetes Res. 2024 Apr 29;2024:2935795. doi: 10.1155/2024/2935795 (PMC11073849; doi:10.1155/2024/2935795)
Supplement: Supporting Information — Additional supporting information can be found online in the Supporting Information section. The extracted data from the included studies. [file 2935795.f1.docx]

**Table 2: Data Extraction Sheet for included studies.**

| Author(S)/ Year of Publications | Country | Purpose Of the Study | Study Design | Study Population | Sample Size | Intervention/ Outcome | Summary of findings. |
| --- | --- | --- | --- | --- | --- | --- | --- |
| [4] | Iran | To investigate the  effect of an educational intervention based on the Trans Theoretical Model (TTM) on the physical activity level of diabetic  patients. | Randomized Control Trial | Type 2 diabetes patients | 120 | Presentation of Educational Content;  1. To increase physical activity, 3 pamphlets containing solutions were provided to participants.  2. To understand the principles of physical activity in diabetic patients, 1 guide manual was given to participants.  3. On the walking exercise program for a diabetic patient, 1 guide manual was given.  4. On how to perform physical activities, 1 CD containing how to perform physical activities suitable for diabetic patients was provided. | The study concluded that education and intervention based on TTM can be effective in increasing the level of physical activity among diabetic patients. |
| [8] | China | To find the effect of the trans-theoretical model (TTM) of behavior change in diabetic kidney disease (DKD) patients treated with maintenance  hemodialysis (MHD) | Experimental research design | Diabetes kidney disease (DKD) patients | 80 | Health Education  1. At the pre-contemplation and contemplation stages,  Patients and caregivers were motivated to recognize the significance of behavior change.  2. At the preparation stage patients were educated via online platforms (WeChat) to change by utilizing the acquired related knowledge in daily life. Also, one-on-one interviews were conducted for caregivers to clarify cognitive bias related to treatment compliance.  3. At the action stage, factors influencing behavioral problems were co-analyzed with patients and their caregivers to cultivate favorable behavior habits.  4. a compliment interview was employed to strengthen the patients’ effective behavior at the maintenance stage. | The study revealed that the nursing method combined with the TTM of behavior change can relieve patients’ bad psychological  state, increase compliance to fluid intake, and  effectively reduce the incidence rate of dialysis-associated  acute complications. |
| [5] | Iran | To show the effects of intervention based on the transtheoretical model on physical activity and metabolic control of diabetic women. | Randomized control trial study | Diabetic women | 98 | Educational contents;  1. Increasing the individuals’ awareness of the disadvantages of lack of physical activity and its advantages.  2. Restatement of  individuals’ positive experience with exercise in the past.  3. Using verbal persuasion, introducing successful individuals among diabetic women  community in physical activity and the level of HbA1c control as a model.  4. Divided goals into help to increase self-efficacy about doing physical  activity and statement of participants ‘experience in each session were used  in educational programs.  5. Finally, a training package was given to the intervention group including a pamphlet, folder, and training CD about exercise in diabetics. | The study concluded that education of diabetics based on the Trans Theoretical model is useful for increasing the self-efficacy of patients.  Again, comprehensive training by increasing physical activities in the long term may be effective in controlling diabetes. |
| [12] | Malaysia | To identify and explore the factors affecting type 2 diabetes mellitus (T2DM) patients'  adherence to lifestyle change recommendations | Cross-sectional survey | Type 2 diabetes mellitus patients | 163 | (1) Health-care providers should  identify patients' stages of change before lifestyle change implementation.  (2) Determining the stages of change and self-confidence of T2DM patients is needed to ascertain long-term  adherence. |  |
| [10] | Ethiopia | To determine predictors of regular physical activity among type 2 diabetes mellitus patients | Cross-sectional study | Type 2 diabetes mellitus patients | 400 |  | The study recommends TTM in designing interventions to increase and maintain physical activity behavior in Type 2 diabetes mellitus patients |
| [11] | India | To determine the predictive power of the transtheoretical model (TTM) of physical activity behavior in patients with type-2 diabetes. | Cross-sectional study | Type-2 diabetes patients. | 393 | . | The study recommended TTM-based interventions to promote and maintain exercise behavior given it effectiveness. |
| [20] | Iran | To determine nutritional behaviors |  | Pre-diabetic patient | 220 |  | Element of the trans-theoretical model is effective in developing health behavior change interventions. |
| [15] | Taiwan | To investigate the key factors that promote behaviour change in diabetic subjects using the  transtheoretical model. | Cross-sectional study | Type 2 diabetes patients | 317 |  | To develop a tailored self-management plan behaviour change strategies should depend on individuals’ stages of behaviour change. This would help the planner to understand the specific demands in depth and the need for a social network. |
| [16] | USA | To describe how Resources and Supports for Self-Management (RSSM) and strategies of the transtheoretical model (TTM) intersect to produce a comprehensive approach resulting in cutting-edge  Diabetes programs. |  |  |  |  | The study demonstrated that the  TTM enhances RSSM and facilitates the adoption of  good diabetes self-management behaviors. |
| [6] | Iran | To determine the impact of education based on the Trans-Theoretical Model on the promotion of  physical activity among children of patients with hypertension and diabetes | Interventional clinical trial research | Children of patients with hypertension and diabetes | 179 | Educational Content (6 months training course);  The case group was trained within three two-hour sessions using different practices. | The intervention program based on the Trans Theoritical Model caused progress  and promotion in physical activity of patients’ children. |
| [21] | Spain | (1) To identify the contribution of the model to interventions in 6 months.  (2) To identify the contribution of the model to interventions in diabetic patients.  (3) To assess whether interventions based on the transtheoretical model are really effective in patients with diabetes. | Bibliographic search | Studies that applied the transtheoretical model to diabetes. | 16 | (1) The use of validated  questionnaires in the assessment process is recommended.  (2) The studies included in this review show how the transtheoretical model has made a positive contribution to interventions in the field of diabetes |  |
| [3] | Iran | To determine:  (1) the behavioral intention or profile of patients with type 2 diabetes mellitus (T2DM) based on the stages of the change model. | Mixed method study | Type 2 diabetes patients | 246 | Self-Management Education;  Four focus group discussions (FGDs) in the field of self-management were organized for participants. The FGD began with a general and open-ended question like “How is life with diabetes?” Afterward, every patient described his/her disease in one way. The next and  pursuing questions were based on the information that the patient revealed. If needed, probing questions were also  asked to achieve the purpose of the study. | This study indicated facilitator and barrier factors in SM based on TTM in  action and pre-action groups. Healthcare professionals should consider these findings to  improve the patients’ outcomes. |
| [17] | Iran | To determine factors related to physical activity based on trans theoretical model among sample of Iranian diabetic patients. | Cross-sectional study | Diabetic patients. | 301 | Physical activity promotion program for diabetic patients  should be focus on increasing self-efficacy toward doing physical activity and attention to behavior process of change. | The study confirmed that TTM was useful for health educator for designing appropriate program for diabetic patients. |
| [19] | China | To investigate the influence of diet intervention based trans-theoretical model on pre-diabetes | Experimental research. | Patients with pre-diabetes | 122 | Diet intervention based TTM and health education related to diabetes were administered for three months and six months, respectively. | The study confirmed that dietary intervention based on TTM can effectively improve knowledge of diabetes diet, behavior of diabetes diet, the quality of life in patients with pre-diabetes. |
| [24] | Iraq | To determine the role of the Trans-theoretical Model in enhancing glucose level-controlling behavior for clients with DM. | Experimental design | Diabetic patients | 60 |  | The study confirmed that the Trans-theoretical Model is effective in enhancing glucose level-controlling behavior in clients with DM. |
| [13] | Turkey | To determine the effect of a transtheoretical model–based  motivational interview method on self-efficacy, metabolic control, and health behavior in adults with type 2 diabetes mellitus. | A randomized controlled study design | Individuals with type 2 diabetes mellitus. | 50 | TTM‐based motivational interview: TTM‐based motivational interviews were performed (using TTM-base motivational interview guides) to assess targets and approaches to nutrition, exercise,  and medication uses behavior stages of the participants in the intervention group according to the Diagnosis Form for Behavioral Change Stage in Patients with Type 2 Diabetes Mellitus. Motivational  interview methods such as expressing empathy, developing discrepancy, rolling with resistance, supporting self‐efficacy, avoiding giving advice, providing simple decisional balance, using an importance‐confidence scale, using open‐ended questions, reflecting, and summarizing were used. | The transtheoretical model-based motivational interview method  increased the self‐efficacy level of participants with type 2 diabetes mellitus, which helped them improve their metabolic control and health behavior stages over this 6‐month period. |
| [14] | UK | To examine the application of a TTM approach to increase physical activity in people with type 2 diabetes by a specialist dietitian. | Randomized controlled trial. | People with  type 2 diabetes. | 40 | Exercise  Consultation Interview (ECI):  The ECI session involved a 20–30 min one-to-one discussion with a dietitian who was trained in TTM-based counseling and motivational interviewing and the provision of physical activity advice. Participants also completed the Health Education Authority’s ‘Helping People Change’ training course. Once physical activity options were established, short-term goals were set and participants were given these in writing. Examples included suggestions such as walking to work or getting off the bus a few stops earlier to increase walking. | The study concluded that a specialist dietitian with motivational interviewing and behavioral change training can successfully deliver a TTM  intervention to people with diabetes that results in an increase in  physical activity and stage of change. |
| [18] | USA | To describe the trans-theoretical model and to discuss strategies for applying it to diabetes management. | Descriptive study | Diabetic patient |  | Self-management skills:  Including regular self-monitoring of blood glucose, taking medications appropriately, eating nutritiously and on schedule, maintaining and achieving a healthy body weight, engaging in regular physical activity, recognizing and managing hypoglycemia and hyperglycemia, practicing appropriate foot care, quitting smoking and dealing with illnesses that arise | Utilizing stage-matched strategies may be beneficial in motivating clients with diabetes to adhere to recommendations. |
| [7] | Saudi Arabia | To examine the effect of the new  behavioral model, the TTM Model short messages (text 4 change) to modify lifestyle to prevent or delay the onset of T2DM, through the promotion of a healthy diet and increased physical activity, in impaired glucose  tolerance patients. | Randomized Controlled Trial | Individuals with type 2 diabetes mellitus | 1016 | All participants receive group education and motivation about lifestyle modifications along with written information about diet and physical activity. All participants took Metformin 500 mg twice daily. | This new approach for promoting behavior modification in pre-diabetics is expected to delay and/or prevent the development of T2DM in Saudi Arabia. |
| [9] | Taiwan | To find out the role of knowledge and stages of change (Soc) as serial mediators linking health literacy to glycemic control. | Cross-sectional survey | People living with type 2 diabetes. | 232 |  | The results help to form a basis for the development of Stages of Change interventions to promote self-management of diabetes through glycemic control. |
